# Painful ophthalmoplegia of the left eye in a 19-year-old female, with an emphasis in Tolosa-Hunt syndrome: a case report

**DOI:** 10.4076/1757-1626-2-8271

**Published:** 2009-09-17

**Authors:** Juan A Mendez, Cristhian R Arias, Diego Sanchez, Luis M Pesci, Brenda S Lopez, Ruben Lopez, Elvira Castro

**Affiliations:** 1 Department of Internal Medicine, Calzada de Tlalpan 4800, Colonia Sección XVITlalpan, C.P. 14080. Distrito FederalMéxico; 2 Department of Ophthalmology, Calzada de Tlalpan 4800, Colonia Sección XVITlalpan, C.P. 14080. Distrito FederalMéxico; 3 Department of Neurology, Hospital, Calzada de Tlalpan 4800, Colonia Sección XVITlalpan, C.P. 14080. Distrito FederalMéxico

## Abstract

**Introduction:**

Painful ophthalmoplegia refers to periorbital or hemicraneal pain plus ipsilateral ocular motor nerve palsies with or without oculo-sympathetic paralysis, sensory loss in the distribution of V1 and V2 can co-occur. There are many etiologies of painful ophthalmoplegia. Tolosa-Hunt syndrome is a steroid-responsive painful ophthalmoplegia secondary to idiopatic granulomatous inflammation of the cavernous sinus or orbital apex. THS is a diagnosis of exclusion and treatment should be with high dose steroid.

**Case presentation:**

We describe the case of a 19-year-old female that was admitted to our hospital for painful ophthalmoplegia of the left eye. After the diagnostic work-up, we concluded that the patient had a benign form of Tolosa-Hunt syndrome. We initiated treatment with steroids and 72 hours later saw a response.

**Conclusion:**

In conclusion, steroid treatment is the cornerstone in the management of THS. Even though there is no standardized dose specified in the literature, this type of treatment with steroids at a dose of 1 mg/kg/day tapered slowly over 3 to 4 months has been well received.

## Introduction

Painful ophthalmoplegia refers to periorbital or hemicraneal pain plus ipsilateral ocular motor nerve palsies with or without oculo-sympathetic paralysis, sensory loss in the distribution of the ophthalmic and occasionally the maxillary division of the trigeminal nerve can co-occur [1]. The only anatomic location where the ocular motor nerves, the first division of the trigeminal nerve and the internal carotid artery co-exist are the cavernous sinus and superior orbital fissure [1]. Painful ophthalmoplegia can result from neoplasic, vascular, inflammatory or infectious disease.

Tolosa-Hunt syndrome (THS), a steroid-responsive painful ophthalmoplegia secondary to idiopatic granulomatous inflammation of the cavernous sinus or orbital apex [2,3], was first described by Tolosa in 1954 [4]. In 1961, Hunt et al [5], reported six cases of painful ophthalmoplegia; that rapidly improved with the use of steroids. THS criteria was first provided by the International Headache Society (IHS) in 1988 [6], and modified (Table 1) in the revised IHS headache classification of 2004 [7]. THS can be classified [8], according to neuroimaging in benign (when no abnormal neuroimaging can be found), inflammatory (when inflammatory findings are shown on MRI or biopsy) and symptomatic (when neuroimaging reveals specific lesion). THS is a diagnosis of exclusion; diagnostic work-up [1], includes routine blood work, inflammatory markers, fasting glucose, CSF evaluation, ANA, anti-dsDNA, c-ANCA, MRI, conventional angiography or MRA; and in some cases biopsy. Treatment should be with high dose steroids (1 mg/kg/d) tapered slowly over 3 to 4 months [1].

**Table 1.** ICHD-II classification part three. Cranial neuralgias, central and primary facial pain and other headaches

*13.16 Tolosa-Hunt syndrome*
Description:
Episodic orbital pain associated with paralysis of one or more of the third, fourth and/or sixth cranial nerves which usually resolves spontaneously but tends to replase and remit.
Diagnostic criteria:
A. One or more episodes of unilateral orbital pain persisting for weeks if untreated
B. Paresis of one or more of the third, fourth and/or sixth cranial nerves and/or demonstration of granulomas by MRI or biopsy
C. Paresis coincides with the onset of pain or follows it within 2 weeks
D. Pain and paresis resolve within 72 h when treated adequately with corticosteroids
E. Other causes have been excluded by appropriate investigations^1^
Note:
1. Other causes of painful ophthalmoplegia include tumours, vasculitis, basal meningitis, sarcoid, diabetes mellitus a ophthalmoplegic ‘migraine’.
Comments:
Some reported cases of Tolosa-Hunt syndrome had additional involvement of the trigeminal nerve (commonly the first; division) or optic, facial or acoustic nerves. Sympathetic innervation of the pupil is occasionally affected.
The syndrome has been caused by granulomatous material in the cavernous sinus, superior orbital fissure or orbit in some biopsied cases.
Careful follow-up is required to exclude other possible causes of painful ophthalmoplegia.

We report the case of a patient with painful ophthalmoplegia of the left eye with emphasis on the Tolosa-Hunt syndrome, its diagnostic work-up and treatment.

## Case presentation

We present the case of a 19-year-old Hispanic female previously healthy, whose relevant medical history only included smoking 4 cigarettes a day for one year. The patient was admitted to our hospital for left periorbital pain, ipsilateral ocular motor nerve palsies and diplopia. Four days prior to admission, the patient started with first episode in her life of severe left periorbital pain; 48 hours later, she also presented limited left eye movements, ipsilateral palpebral ptosis and horizontal diplopia. Pain did not cede after the administration of NSAID, which was the reason why the patient decided to resort to the ER for examination.

**Figure 1.**
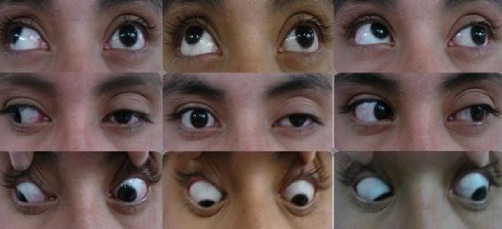
Neuro-ophthalmologic examination prior treatment shows left palpebral ptosis, exotropia of the primary look of the left eye, paresis of the third, fourth and sixth left cranial nerves.

At the hospital, she was submitted to a thorough physical examination by a multidisciplinary team integrated by three ophthalmologists, a neurologist and three specialists in internal medicine. The results of the physical and neuro-ophthalmologic examination were weight 89 kg, height: 1.57 mt, BMI 36 kg/m^2^, 20/20 vision, normal eye fondues bilaterally, left palpebral ptosis, exotropia of the primary look of the left eye, paresis of the third, fourth and sixth left cranial nerves, and hypoesthesia over the first and second division of the left trigeminal nerve. The right eye and the rest of the physical examination did not show further abnormalities (Figure 1).

Since the beginning, we considered Tolosa-Hunt syndrome as a possibility, but started the clinical approach as a painful ophthalmoplegia. Her initial laboratory tests showed white blood cell count, 8.500/ml; red blood cell count, 4.86×10^6^/μl; haemoglobin, 15.1 g/dl; platelets, 293×10^3^; glucose, 98 mg/dl, blood urea nitrogen, 8 mg/dl; creatinine, 0.6 mg/dl; ELISA for HIV, negative; D-Dimer, 271 ng/ml (<500 ng/ml). Thyroid function tests showed TSH, 1.00 μUI/ml (0.34-5.60); Total T3, 0.96 ng/ml (0.87-1.78); free T 3, 2.64 pg/ml (2.50 y 3.90); free T4, 0.76 ng7 dl (0,54-1.64); Total T4, 10.72 μg/dl (6.09-12.23). The cerebral spinal fluid reported 2 mononuclear cell/uL; glucose, 51 mg/dl; proteins, 15 mg/dl; ADA and PCR in CFS for tuberculosis and cultures were negative. ANA´s, were positive in a homogenous pattern 1:40; anti-dsDNA, 15.1 U/ml (0-9.6); c-ANCA, positive 1:40 and x-ANCA, 1:20. CT scan of brain and paranasal sinus, MRI and MRA of the brain were normal.

**Figure 2.**
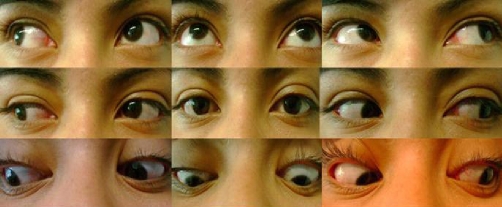
Neuro-ophthalmologic examination after treatment shows improvement of the left palpebral ptosis and left eye movements after 72 hours of steroid treatment.

Since the studies showed no abnormalities and we excluded neoplasic, infectious, vascular, thyroid and metabolic causes of painful ophthalmoplegia, we decided to start treatment for Tolosa-Hunt Syndrome with metilprednisolne 1 gr IV daily for 3 days, and noticed significant response of the left periorbital pain, palpebral ptosis and the ipsilateral ocular motor nerve palsies in the next 72 hours. At day 4, we changed treatment to prednisone at 1 mg/kg daily. One week later, we initiated steroid tapering every week, and at week 6 the patient was asymptomatic and her neuro-ophthalmologic examination was completely normal. Today the patient is still on prednisone 10 mg/day (Figure 2).

## Conclusion

We concluded that the patient had Tolosa-Hunt syndrome because she completely fulfilled HIS 2004 diagnostic criteria, and since no abnormalities were found in her laboratory tests and neuroimaging, we classified her as a benign variety of Tolosa-Hunt syndrome. ANA’s, anti-dsDNA and ANCA’s were positive but we considered them too “weak” for the diagnosis of a painful ophthalmoplegia secondary to a vasculitis such as Wegener or Lupus because our patient had no other clinical or laboratory finding that supported diagnostic criteria for these two diseases.

We would also like to emphasize the importance of steroid treatment; even though there is no standardized dose indicated in the literature, this type of treatment with steroids at a dose of 1 mg/kg/day tapered slowly over 3 to 4 months has been well received.

## Abbreviations

ADA: adenosine deaminase
ANA: antinuclear antibody
ANCA: anti-neutrophil cytoplasmic antibodies
CSF: cerebrospinal fluid
CT: computerized tomography
ER: emergency room
IHS: international headache society
MRA: magnetic resonance angiography
MRI: magnetic resonance imaging
NSAID: non-steroidal anti-inflammatory drugs
PCR: polymerase chain reaction
TSH: Tolosa-Hunt syndrome
